# Evolutionary Divergent Suppressor Mutations in Conformational Diseases

**DOI:** 10.3390/genes9070352

**Published:** 2018-07-13

**Authors:** Noel Mesa-Torres, Isabel Betancor-Fernández, Elisa Oppici, Barbara Cellini, Eduardo Salido, Angel L. Pey

**Affiliations:** 1 Department of Physical Chemistry, University of Granada, 18010 Granada, Spain; noelmesatorres@gmail.com; 2 Hospital Universitario de Canarias, Center for Rare Diseases (CIBERER), University of La Laguna, 38320 Tenerife, Spain; ibetfer3@gmail.com (I.B.-F.); edsalido@gmail.com (E.S.); 3 Department of Neurosciences, Biomedicine and Movement Sciences, Section of Biological Chemistry, University of Verona, 37134 Verona, Italy; elisa.oppici@gmail.com; 4 Department of Experimental Medicine, University of Perugia, 06132 Perugia, Italy; barbara.cellini@unipg.it

**Keywords:** protein stability, conformational diseases, disease-mechanisms, compensatory mutations, molecular therapies, genotype-phenotype correlations

## Abstract

Neutral and adaptive mutations are key players in the evolutionary dynamics of proteins at molecular, cellular and organismal levels. Conversely, largely destabilizing mutations are rarely tolerated by evolution, although their occurrence in diverse human populations has important roles in the pathogenesis of conformational diseases. We have recently proposed that divergence at certain sites from the consensus (amino acid) state during mammalian evolution may have rendered some human proteins more vulnerable towards disease-associated mutations, primarily by decreasing their conformational stability. We herein extend and refine this hypothesis discussing results from phylogenetic and structural analyses, structure-based energy calculations and structure-function studies at molecular and cellular levels. As proof-of-principle, we focus on different mammalian orthologues of the NQO1 (NAD(P)H:quinone oxidoreductase 1) and AGT (alanine:glyoxylate aminotransferase) proteins. We discuss the different loss-of-function pathogenic mechanisms associated with diseases involving the two enzymes, including enzyme inactivation, accelerated degradation, intracellular mistargeting, and aggregation. Last, we take into account the potentially higher robustness of mammalian orthologues containing certain consensus amino acids as suppressors of human disease, and their relation with different intracellular post-translational modifications and protein quality control capacities, to be discussed as sources of phenotypic variability between human and mammalian models of disease and as tools for improving current therapeutic approaches.

## 1. Single Amino Acid Changes as Key Players in Molecular Evolution, Human Physiology and Genetic Diseases

Life relies on the function of proteins to carry out biochemical reactions and control metabolic pathways. The pioneering work by Christian Anfinsen [1] showed that the process by which a protein acquires its folded and functional state (protein folding) can be spontaneous, and since then, the linkage between protein amino acid sequence, tridimensional structure and function has become a paradigm in Biology [2]. Although the folded and native state is one of low free energy (thus stable), protein folding inside the cell is often inefficient due to the existence of pathways competing with native folding (e.g., aggregation) that may compromise protein function and cellular homeostasis [2]. To circumvent these issues, life has developed complex intracellular machineries to assist protein folding and to control protein’s life cycle, including subcellular transport and eventually protein degradation (collectively referred to as the protein homeostasis network [2,3]). Naturally, the amino acid sequence of proteins can vary within a species with impact in their folding, stability and function, due to post-translational modifications (PTMs), and inherited non-synonymous (missense) mutations. These alterations have important implications to understanding human physiology, molecular evolution and genetic diseases [2,3,4,5].

Despite the complex biological mechanisms available to ensure DNA replication and repair, mutations are inherent to life [6]. Studies aimed at understanding the mechanisms by which mutations happen and prevail within existing populations of the species and between species along evolutionary time scales (in the order of millions of years, Myr) have supported the existence of complex mutational dynamics [6,7,8,9,10]. From a very simple perspective, mutations can be classified in three main groups according to their effects on function, phenotype, and organism fitness: (i) adaptive, those that may provide some beneficial effect on function (for instance, improving protein stability, function or regulation) and phenotype, thus increasing fitness; (ii) neutral, as those that have no apparent beneficial effect in molecular properties, phenotype or fitness but may act as potential seeds for novel functions or adaptive traits; (iii) deleterious, often those drastically affecting molecular properties and fitness [6,11,12,13]. Adaptive and neutral mutations are thought to be often fixed along evolution, while very deleterious mutations are usually purged [6,11]. Although illustrative, this way of cataloguing mutations obviously depends on the way we define and interpret mutational effects on function, phenotype and fitness [6]. First, proteins are often capable of carrying out multiple functions, acting as catalysts of different biochemical reactions and developing regulatory protein:protein and protein:nucleic acid interactions [14]. Consequently, mutational effects on molecular functionality are multi-featured variables that should be ultimately regarded in particular genomic, proteomic and cellular contexts [15]. Second, a phenotype depends on the “penetrance” of the changes in molecular functionality into biological function, which may be influenced by multiple regulatory mechanisms including inter-individual and inter-species differences in protein homeostasis networks [16]. Third, fitness is a continuous variable that ultimately relates the effect of a given mutation on the reproductive capacity of the organism compared to a wild-type sequence [6]. When we think about these features regarding the molecular consequences of mutations along evolution and in human disease, we may reach an intriguing idea: while most deleterious mutations are not fixed along evolution, some of these “selfish” mutations may survive in human population as long as patients can have offspring despite the outcome of disease, as well as due to some adaptive side-effects (e.g., increased resistance towards infectious diseases; [17,18]). Interestingly, disease-associated mutations in human genes are quite often found as wild-type residues in other mammalian species [10,19,20], suggesting certain vulnerability of some human proteins towards conformational disease. These ideas also suggest that some neutral mutations may have been recently fixed in an evolutionary time scale, for instance trading off stability and regulation (at the enzyme activity or interactomic levels) adaptively, but rendering human proteins more vulnerable towards disease-associated missense mutations than other mammalian orthologues. Along this manuscript, we will discuss some recent work suggesting that this might be the case for some human proteins and genetic diseases, representing an example of epistasis (“context dependence of mutational effects”; [19]). Therefore, pathogenic (i.e., deleterious) mutations in human genes could be considered as neutral in other mammalian sequences due to the presence of compensatory mutations often in their spatial proximity [10,20], although long-range interactions have also been recently described [21]. From the study of these compensatory mutations in the presence of disease-associated mutations, insight can be gained into: (i) the mechanisms of disease and novel therapeutic approaches, (ii) how mutations affect multiple functional and structural sites along molecular evolution, and (iii) potential roles of protein stability and dynamics in protein evolution and human disease.

## 2. Control of Protein Function and Life Cycle through Structural Stability and Dynamics

In vitro conformational stability of proteins is often discussed from two different but intrinsically related view points, thermodynamic and kinetic ones. Thermodynamic stability refers to the unfolding free energy change between native and non-native states (e.g., the unfolded state), which dictates the fraction of the protein populating each of these states. Kinetic stability refers to the denaturation rate, and implicitly to the time scale in which the protein remains in the folded and active conformation [22,23]. Consequently, protein thermodynamic and kinetic stability are generally thought to be adequate for the environmental conditions in vivo at which they must operate (also considering that conformational stability is further modulated by the level of substrates, the occurrence of PTMs, protein:protein interactions…). For instance, thermodynamic and kinetic stabilities correlate well, within extant protein orthologues, with the optimal growth of the organism, and accordingly, conformational stability has also changed in parallel with the Earth’s temperature across geological time scales [24,25]. However, it is not clear to what extent thermodynamic/kinetic stability determines the rate of degradation, for instance in eukaryotic cells, and thus, whether or how the effects of disease-associated mutations on conformational stability translate into relevant degradation rates. In this context, to understand the life cycle of mammalian proteins and the effects of disease-associated mutations, it is important to take into account protein local stability and dynamics as well as PTMs such as ubiquitylation and critical-to-determine protein degradation in eukaryotes [26,27,28].

The two main mechanisms involved in the degradation of proteins are the ubiquitin-dependent proteasomal (UDP) and the lysosomal autophagy pathways. The former is involved in the degradation of over 80% of all proteins, and fundamentally recognizes folded, misfolded, and damaged proteins, while the latter mostly clears large aggregated structures [29,30]. Both pathways are linked to PTMs through ubiquitylation of protein substrates as the main degradation signal [29]. For UDP degradation, a minimal tri-partite model has been proposed, in which different degradation signals (degrons) must exist in a substrate to be degraded: (i) a primary degron, a linear motif that binds to ubiquitin E3 ligases and mediate ubiquitylation by conjugating E2 enzymes; (ii) a secondary degron close to the primary degron where one or several lysine residues are mono- or poly-ubiquitylated; (iii) a tertiary degron, acting as initiation site for degradation by the 26S proteasome [27,31]. Importantly, these three types of degrons are preferentially found in dynamic or partially unstructured regions [27,32]. Consequently, while a certain degree of conformational stability must exist for a protein to be active, the determinants of their intracellular degradation rate may reside in local structural stability and dynamic features in regions amenable for ubiquitin tagging. Although the situation in vivo is likely more complex [29,32], these concepts will be particularized and shown to be critical for cancer-associated NADP(H):quinone oxidoreductase 1 (NQO1) (Section 3 and Section 4).

To understand the roles of mutations in evolution and development of disease, it is important to remark that the native state of proteins is more adequately described by an ensemble of conformations (native state ensemble), in which a variety of structure-encoded protein motions occur [13,33]. Protein dynamics is intimately linked to their function, regulation, degradation, and evolution [13,33,34]. Missense mutations may cause dramatic effects on protein function and regulation through modulation of protein dynamics [13,35], and the critical role of dynamics in enzyme evolution is becoming well established [36,37,38]. However, how disease-associated missense mutations cause loss of function linked to alterations in protein dynamics, and the modulation of their effects by interaction with compensatory mutations or PTMs have been rarely addressed.

## 3. Mutational Effects in Loss-of-Function Disease Mechanisms

**Figure 1:**
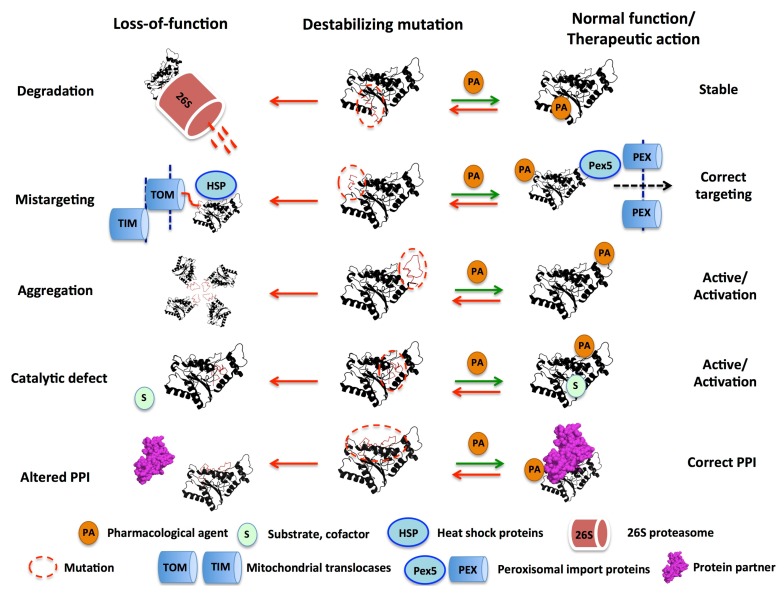
Schematic representation of five of the main molecular mechanisms of loss-of-function (LOF) diseases associated with destabilizing mutations and relevant examples discussed in this review: (i) accelerated protein degradation by the 26S proteasome (e.g., the polymorphism P187S in NQO1; see Section 4.1.); (ii) intracellular mistargeting to mitochondria due to enhanced interaction with mitochondrial import machineries instead of normal targeting to peroxisomes upon interaction with Pex5p dependent pathway (e.g., variant G170R of AGT associated with PH1; see Section 4.2.); (iii) enhanced formation of inactive aggregates (e.g., variant I244T of AGT associated with PH1; see Section 4.2.); (iv) catalytic defect (e.g., the polymorphism P187S in NQO1 promotes the formation of the inactive apo-enzyme by altering FAD binding; see Section 4.1.); (v) alteration in protein:protein interactions (e.g., the polymorphism P187S reduces NQO1 steady-state levels thus destabilizing transcription factors such as p53 and p73α; see Section 4.1.). On the right side, the potential correction of local destabilizing effects by a pharmacological agent (e.g., a small ligand) is presented as promising approaches to rescue the function of disease-associated variants. AGT, Alanine:glyoxylate aminotransferase; PH1, primary hyperoxaluria type I; NQO1, NADP(H):quinone oxidoreductase 1; PPI, protein:protein interaction.

Human genetic diseases are often caused by missense mutations, which are classified according to their molecular phenotype or pathogenic effects in two large groups: loss-of-function (LOF), in which the mutation ultimately impairs the activity of the protein, or gain-of-function (GOF), where the mutations affect regulation of the protein [39] or provide a toxic function to the protein (such as in amyloidosis) [2]. Along this review we will primarily focus in LOF diseases, in which pathogenic consequences are caused through different mechanisms (the most relevant are depicted in Figure 1). Although easy to describe conceptually, the precise molecular effects of mutations leading to disease through one (or several) of these mechanisms are challenging to be known. Understanding the specifics of these mechanisms at the molecular level is important not only for the basic research, but also to develop efficient therapies (for instance, pharmacological chaperones; [40]) aimed at targeting molecular alterations without compromising normal regulatory or functional properties (Figure 1). Intuitively, some lessons to target these alterations can be learned from studying the mechanism of action of suppressor or compensatory mutations [21,41].

Traditionally, the understanding of disease mechanisms has arisen from characterizing certain molecular properties of purified proteins, analyzing suitable structural models, and by correlating these effects with abnormal behavior in cellular or animal models. These approaches have worked well so far with mutations affecting key residues in catalytic sites or oligomerization interfaces, or those causing other devastating effects on structure and stability. However, proteins have complex behaviors at multiple levels. For instance, how a certain mutation can simultaneously affect multiple functions in a given protein (often referred to as “pleiotropy”) is difficult to rationalize from the inspection of structural models (which are customarily performed using wild-type crystal structures). In addition, these models rarely provide information on the mutational effects on protein dynamics. While mutational effects on protein stability can be estimated either by experiments (a thermal denaturation assay) or calculations (for instance, the algorithm FoldX), they do not shed light into how destabilization percolates through the protein structure, affecting different functional sites. This might be a key issue since destabilizing effects can propagate to distant sites in the protein structure [42], thus providing a structural and energetic framework to explain how mutations far from binding, regulatory, or catalytic sites can affect different functional sites in non-trivial manners [35,43].

**Figure 2:**
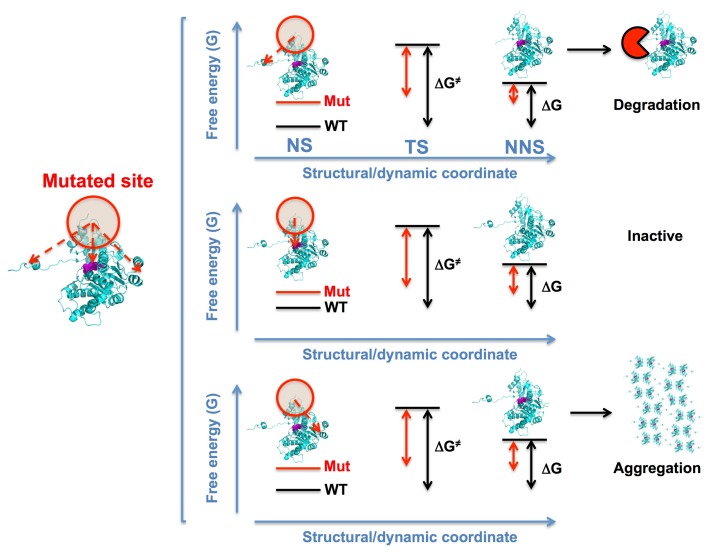
Simple models to describe the potential impact of a disease-associated mutation in different pathogenic traits (degradation, inactivation, aggregation) through long-range effects. The propagation of destabilizing effects to distant functional/structural sites associated with each mechanism can lead to quantitatively different roles of the mechanisms in the pathogenesis. The first shell of interactions is shown as a red and shadowed circle while propagation to distant site is shown by red arrows. NS, native state; TS, transition state; NNS, non-native state involved in the pathogenic mechanism. For sake of illustration, the mutation primarily affects the stability (free energy) of the NS with little effects on the TS and NNS, thus leading to thermodynamic and kinetic destabilization regarding that particular pathogenic mechanism. In addition, the mutation is displayed as preferentially promoting a particular pathogenic trait (in this case, degradation vs. inactivation or aggregation). Changes in free energy levels upon mutation should not be considered to provide fully quantitative effects. Mut, mutant; WT, wild type.

To introduce some of these aspects in a semiquantitative manner, we will use a simple conceptual framework (Figure 2). This is exemplified by showing the effects of a mutation which causes destabilization and/or dynamic alterations, although it could similarly be used to explain the effect of compensatory mutations or PTMs. For the sake of illustration, the effects in three common LOF mechanisms are displayed: accelerated degradation, aggregation, and enzyme inactivation, although it could likewise capture the effect on other mechanisms shown in Figure 1. The destabilizing effect of the mutation primarily affects the local environment (so-called first shell), and its effect should dissipate through the structure with an effective radius in the range 5–10 Å [42]. Through this dissipation, the mutation may affect the local stability and dynamics (without necessarily changing the structure [42]) beyond the first shell, thus affecting distant sites (up to 20 Å away from the mutated site [42]). In this context, mutational effects could be “sensed” even at distant sites, depending on the magnitude of the original destabilizing effect, the distances between the mutation and the functional sites, and the protein conformational landscape. From this simple approach, several interesting relationships with disease mechanisms (and their correction by, for instance, compensatory mutations or pharmacological chaperones) can be intuitively seen (Figure 2). In general, we assumed for all the mechanisms that the folded protein (NS, native state) exists in equilibrium with certain non-native states (NNS): the equilibrium population between these states is dictated by their free energy difference (ΔG) while the rates of interconversion are determined by the height of the free energy barrier (ΔG^≠^, for the NS). The population of NNS determines the flux of protein molecules towards a certain pathogenic destination (degradation, aggregation, inactivation). When the mutational effects propagate to a region relevant for a pathogenic mechanism (e.g., a region structured in NS, but not in transition state (TS) or NNS), the mutation will increase the flow of protein through the pathogenic pathway either by decreasing the denaturation free energy barrier (and thus, the kinetic stability of the NS) and/or the denaturation free energy difference (thus increasing the population of the kinetic relevant NNS through thermodynamic destabilization of the NS). Importantly, the population of NNS must not necessarily be large to alter intracellular activity or stability, as long as the NNS are those states kinetically relevant and the pathogenic pathway resembles an irreversible process [44,45]. For instance, for UDP degradation, the only requirement would be that the mutation affects the dynamics of a degron (e.g., the initiation site) or enhances the recognition by ubiquitin ligases, accelerating the rate of ubiquitin tagging and thus UDP degradation (here, note that degradation can be considered as irreversible). For LOF aggregation, local destabilization and enhanced dynamics of a region involved in the formation of aggregation-prone species would dramatically accelerate the decay of active protein. For catalytic inactivation, the existing equilibrium in the native ensemble between active and inactive forms (for instance, between ligand binding competent and non-competent states, which is the basis of allostery; [34,46]) can be shifted towards inactive conformations due to structural/dynamic destabilization of the active forms, thus leading to low activity or affinity for substrates or cofactors [21,47]. We will use these models as a simple framework to describe the recent work performed in two model cases involving disease-associated and evolutionary-divergent compensatory mutations.

## 4. NADP(H):quinone Oxidoreductase 1 and Alanine:glyoxylate aminotransferase as Models of Genetic Diseases

### 4.1. NADP(H):quinone Oxidoreductase 1

NQO1 is a dimeric, multifunctional stress protein, involved in the FAD-dependent two-electron reduction of quinones, activation of cancer pro-drugs, and stabilization of transcription factors such as p73α and p53 upon binding [48]. NQO1 monomers are formed by two domains, a large N-terminal domain (NTD, residues 1–224) which binds the cofactor and autonomously form dimers, and a small C-terminal domain (CTD, 225–274) which is required for NADH and substrate binding [48,49]. A common cancer-associated polymorphism, P187S, decreases protein activity and levels in cancer cells by enhancing NQO1 degradation by the UDP. Due to its low steady-state levels, the polymorphism also prevents binding to and stabilization of transcription factors [50,51].

**Figure 3:**
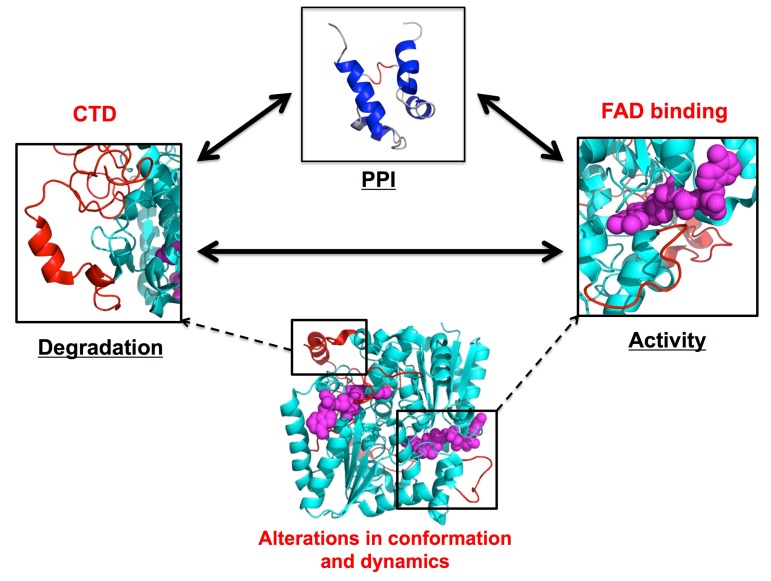
Allosteric network and dynamic communication between functional sites in NQO1 is perturbed by P187S. Three functional sites, the C-terminal domain (CTD), the FAD binding site, and the interaction site with transcription factors, are depicted, of which only the first two were strongly affected by P187S. Please, note that the region of NQO1 involved in PPI is not known, and thus, not highlighted in the figure. Double-headed arrows indicate the proposed communication between functional sites in NQO1 that is perturbed to different extents by P187S. The FAD molecule is displayed as magenta sphere representation, while those regions in the CTD and the FAD binding site perturbed at structural and dynamic levels by P187S are shown as red cartoon representation.

NQO1 is an excellent model to investigate the disease-associated propagation of mutational effects to multiple functional sites according to the models depicted in Figure 2, and particularly, to study the role of protein dynamics in the communication between these sites (Figure 3). The crystal structure of P187S has not revealed noticeable differences in conformation [52], which may suggest that the structure of the NS is hardly affected by this polymorphism. Although the mutated site is located far from the FAD or NADH binding sites, the structural destabilization caused by P187S propagates efficiently to these sites affecting their dynamics, and displaying some degree of communication between them (Figure 3). P187S decreases by 10–50 fold the affinity for FAD [50,53], an effect which is originated from dynamic perturbations of the FAD binding sites and its surroundings (particularly in the loop 58–67) in the apo-state (without FAD bound; note that this state has never been crystallized [54]) (Figure 3). Consistent with ensemble models of allostery, the dynamic destabilization of the FAD binding site would primarily shift the equilibrium in the apo-state towards binding non-competent states (the NNS in the middle panel of Figure 3; [47]). P187S also destabilizes the CTD even in the holo-state (with FAD bound), which remains partially unfolded and highly dynamic in solution [52,54]. The CTD of P187S becomes structured upon binding of the inhibitor dicoumarol [54,55], which is the only form of the full-length polymorphic variant crystallized so far [52]. Altered conformation and dynamics of the CTD are associated with enhanced ubiquitylation and accelerated UDP degradation (which are prevented upon binding of dicoumarol) [54,56,57].

In addition, the communication of dynamic information between functional sites in NQO1 (through an “allosteric network”, Figure 3) can be perturbed by disease-causing missense mutations or deletion of the CTD [21,55]. The deleterious effects of P187S on the FAD binding site (but not in the interaction site with transcription factors) was shown to depend on the presence of the partially unstructured CTD. The communication between different domains and functional sites was further confirmed, as we will discuss later, when compensatory mutations acting as suppressors of P187S phenotype were investigated (Section 5).

### 4.2. Alanine:glyoxylate aminotransferase

Alanine:glyoxylate aminotransferase (AGT) is a dimeric, pyridoxal-5´-phosphate (PLP)-dependent enzyme that catalyzes the transamination of l-alanine and glyoxylate to form pyruvate and glycine [58]. Structurally and functionally, each monomer can be divided in three regions: a short and extended N-terminal tail (NTT, residues 1–21) that grabs the monomers in the dimer, an N-terminal domain (NTD, residues 22–282) containing the active site and most of the dimerization interface, and a C-terminal domain (CTD, residues 283–392) containing the peroxisomal targeting sequence [59]. Human AGT activity is essential for detoxification of the metabolic intermediary glyoxylate in liver peroxisomes, preventing subsequent formation of oxalate and disease development [58,60]. AGT subcellular location varies among mammals, possibly as a reflection of evolutionary origins in metabolic partitioning between different subcellular organelles [61,62,63,64]. There are over 200 mutations in the *AGXT* gene associated with a rare disease (primary hyperoxaluria type I, PH1) inherited in an autosomic recessive manner, in which patients accumulate oxalate that eventually causes renal failure and premature death [58,60]. The *AGXT* gene exists as two polymorphic variants, the most frequent, named as major allele (or wild type (WT)), and a less frequent minor allele (or LM), which carries two single amino acid changes (P11L and I340M) [58]. Although the minor allele is not pathogenic itself, it exacerbates LOF due to additional mutations, thus raising its frequency from about 0.2 globally to 0.5 in PH1 patients [65].

Although the mechanisms by which PH1 mutations cause AGT LOF are diverse, two of them have emerged as very common and particularly interesting for discussion here: aggregation and mitochondrial mistargeting [60,66]. Both are rooted in an enhanced tendency of the protein to populate NNS and altered interaction with the proteostasis machinery upon mutation [60,66,67], which can be thus framed into the mechanisms depicted in Figure 2. However, in contrast to NQO1, in which the altered structure and dynamics of certain functional sites can be blamed to cause a given pathogenic effect (Figure 2), such knowledge is not currently available for aggregation and mistargeting of PH1-causing mutants. Nevertheless, the P11L polymorphism is known to strongly accelerate protein denaturation in the apo-state (without PLP) increasing the population of NNS, effects which are strengthened by PH1 mutations associated with aggregation (e.g., I244T) and mistargeting (the most common, G170R) [67,68,69,70]. This increased population of NNS could explain the enhanced interaction of missense variants with molecular chaperones (Hsp 40, 70, 90, and 60) and their increased aggregation propensity [60,66,67,68,69,71]. In this regard, the N-terminal region of AGT seems to protect towards misfolding [72], a role likely perturbed by the P11L substitution at structural and dynamic levels [67]. Since mutations preferentially leading to aggregation and mistargeting share certain molecular alterations, the mechanistic details underlying one or the other effect are not well understood, although our ongoing work suggests that mutations may differ in the way they affect distant structural features according to the scenarios depicted in Figure 2. It is also apparent that protein homeostasis machineries can handle differently the flow of protein through these two pathogenic pathways, explaining to some extent the finding of some shifting between mechanisms depending on the particular expression conditions [69,73].

## 5. Evolutionary Divergence in Key Compensatory Consensus Amino Acids and its Potential Role in Species-dependent Disease Penetrance

Originally, we were interested in identifying stabilizing mutations for disease-associated proteins such as NQO1 and AGT to generate more active/stable proteins for therapeutic applications (enzyme-replacement and/or nucleic acid therapies) and to identify compensatory/suppressor mutations of disease-phenotypes for pharmacological therapies (e.g., structural hot-spots to drive small molecule screening procedures). To do so, we carried out sequence-alignment (consensus) analyses using sets of AGT or NQO1 proteins from mammalian and/or eukaryotes. After probing the suitability of this approach to make more robust AGT enzymes for therapies [74,75] and to identify disease suppressors for NQO1 [21,47], we realized that divergence at certain sites from the consensus state may have made some (at least, these two) human proteins more vulnerable towards disease-associated mutations (in the context of mechanisms depicted by Figure 1 and Figure 2).

### 5.1. Alanine:glyoxylate aminotransferase

**Figure 4:**
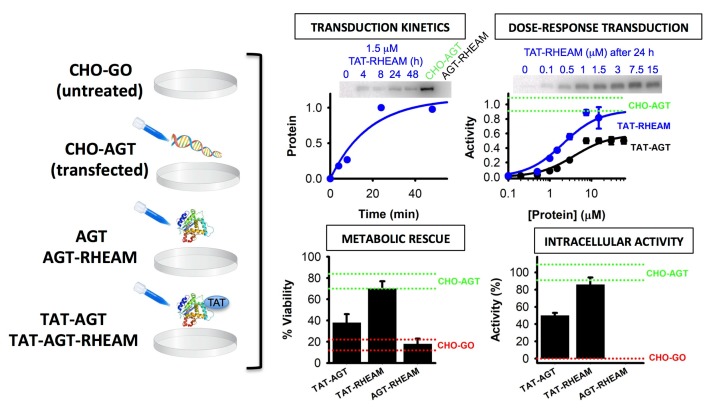
Improved therapeutic correction by consensus AGT-RHEAM in a cell model of enzyme replacement (ERT). CHO-GO cells, which represent a model of PH1, were incubated with fusion proteins made up of a TAT sequence (that confers cell-penetrating ability) attached to the N-terminus of AGT or AGT-RHEAM (TAT-AGTs). Stably transfected CHO-GO cells (CHO-AGT) were used as positive controls, while untransfected cells (CHO-GO) and cells treated with AGT without the TAT peptide (AGT) were used as negative controls. Panels show kinetics of transduction for TAT-AGT-RHEAM (measured by western-blot), dose-response curves for TAT-AGT and TAT-AGT-RHEAM (measured by western-blot and % of specific activity with respect to CHO-AGT cells), metabolic rescue (measured by an indirect glycolate toxicity assay), and intracellular AGT activity expressed as % with respect to CHO-AGT cells. Experiments were performed essentially as described in [76]. Experimental details can be found in Appendix A.

Consensus analyses and subsequent biochemical and biophysical characterization identified five single consensus mutations (Q23R, S48H, D52E, V113A and I340M) that increased the stability of AGT WT in vitro without perturbing its activity [74]. Sequential introduction of these compensatory mutations led to a gradual (and nearly additive) increase in stability, enhancing by three orders of magnitude the kinetic stability in a quintuple variant (Q23R/S48H/D52E/V113A/I340M; abbreviated as RHEAM) in vitro. Further characterization of AGT-RHEAM also revealed increased enzyme activity (about 2.5 fold higher), in particular due to the presence of the S48H mutation [74]. Crystallographic studies showed that particularly two mutations, Q23R and D52E, strengthened a favorable electrostatic interaction network likely responsible for the enhanced in vitro stability [74]. These consensus mutations led to a decrease in population of NNS as well as reduced its aggregation tendency [67]. Importantly, this improvement in activity and stability had no deleterious effects on the expression and targeting of AGT-RHEAM to peroxisomes in cultured cells [74]. Additional studies using different cellular and animal models of disease (Figure 4, Appendix A and [75]) have confirmed the great potential of using consensus mutations to improve enzyme-replacement (ERT) and nucleic acid therapies for rare metabolic diseases.

**Figure 5:**
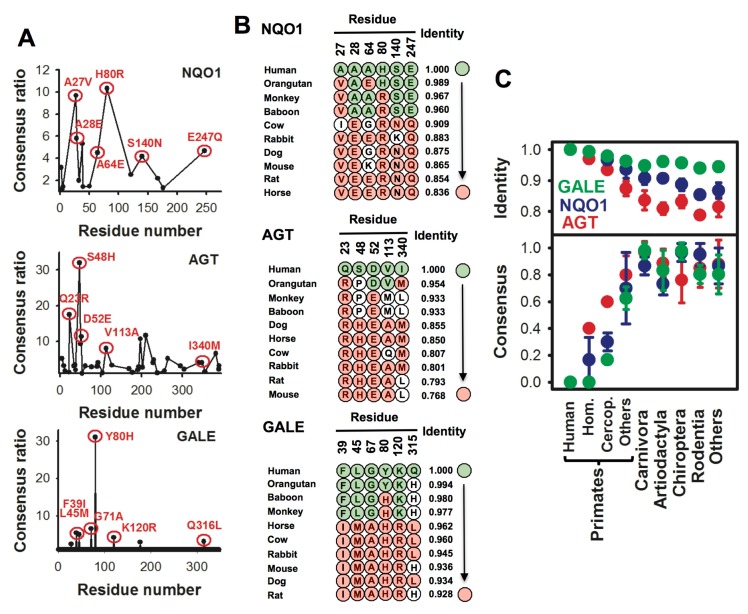
Divergence of consensus amino acids during the evolution of mammalian NQO1, AGT and GALE (uridine diphosphate glucose 4-epimerase): (**A**) Consensus analyses performed using a set of 50 mammalian sequences. Consensus ratio referred to the ratio between the number of sequences in the alignment containing the consensus amino acid and the number of sequences containing the amino acid found in the human protein at that position; (**B**,**C**) Divergence of the set of consensus amino acids; in panel B, divergence is shown for individual sequences, in panel C as grouped by in orders/families (mean ± standard deviation (SD)). Identity referred to pairwise identity comparison of a given sequence with the human protein as query (i.e., the human sequence equals to 1). Data for AGT and NQO1 are from [21] and reproduced with permission.

**Figure 6:**
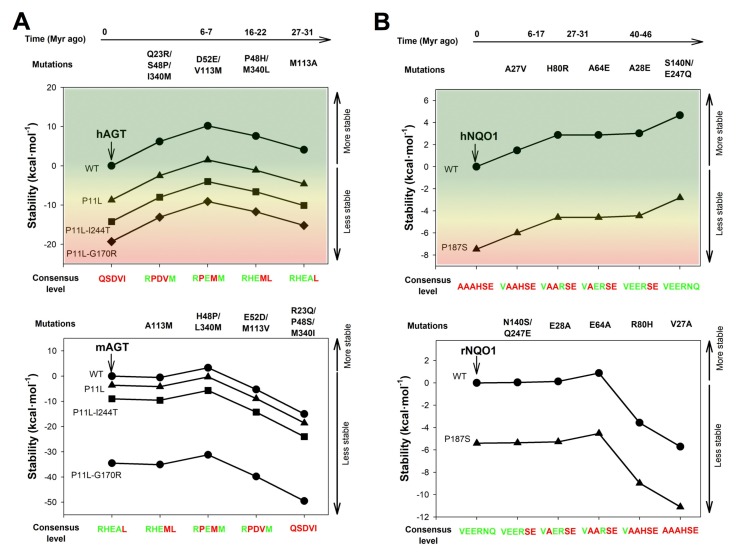
FoldX calculations support the potentially different sensitivity of rodent and human AGT (**A**) and NQO1 (**B**) against disease-associated mutations due to divergence in consensus amino acids. The x-axis shows the sequence of mutations in an evolutionary time-scale for human enzymes as assessed from phylogenetic analyses [21] as well as the level of consensus divergence (divergent, red; consensus, green). Mutational effects on native state stability were evaluated using the FoldX energy field [77] using the structures of human AGT (hAGT: 1H0C), mouse AGT (mAGT: 1QRD) and human NQO1 (hNQO1: 2F1O) and rat NQO1 (rNOQ1: 3KGX). Changes in stability (or ∆∆G) between a mutant and a reference are shown (references are human or rodent enzymes, including in some cases disease-associated missense variants as indicated in the figure). In this way, a positive value in ∆∆G (*y*-axis) shows a gain of stability due to the missense variant. See Appendix B for additional details.

As consensus states along mammalian sequences, the amino acids introduced to generate AGT-RHEAM are commonly observed in non-human sequences. For instance, rabbit and horse AGTs contain RHEAM sets of amino acids, while rat and mouse contain RHEAL (here, I340L is found to bear the silver medal as consensus, with a frequency slightly lower than that of I340M) (Figure 5). Phylogenetic analyses showed that divergence at these sites from the consensus state occurred sharply during primate evolution, within the last 30 Myr (Figure 5 and [21]). The availability of a crystal structure for mouse AGT allowed the identification of a significant degree of conservation of the stabilizing effect of certain consensus amino acids in the rodent enzyme [21]. Therefore, since this reversal to the consensus state at these five sites made human AGT a more robust enzyme, we could think that mouse AGT should also be more robust than the human enzyme by default, for instance, against misfolding caused by PH1 mutations. This hypothesis was further supported by structural comparison of human and mouse AGT [21] and stability predictions performed using the FoldX algorithm (Figure 6A and Appendix B). Addition of consensus amino acids in a sequence that follows a plausible evolutionary trajectory, showed that consensus amino acid could significantly counterbalance the effect of the P11L polymorphism and the two most common PH1 mutations associated with misfolding on the conformational stability of AGT (Figure 6A). Conversely, consensus amino acids could provide a significant stability buffer to mouse AGT against some PH1 associated mutations (Figure 6A). It is worth noting that these FoldX calculations also suggest that certain PH1 mutations might affect more strongly the conformational stability of mouse AGT than that of human AGT (particularly, G170R, see Figure 6A), which may constitute another case of epistatic interaction. Nevertheless, we must bear in mind that these insightful calculations likely reflect the primary impact of the mutations on its local environment, and thus, do not allow to infer information on how these stability changes propagate to those sites involved in aggregation and mistargeting phenotypes (in line with the scenarios depicted in Figure 2) or how non-local epistatic interactions may arise. Unfortunately, how and whether these consensus amino acids may protect human AGT against aggregation and mistargeting, and whether their withdrawal in mouse AGT may render this enzyme more susceptible against PH1 causing mutations inside the cell, remain yet unexplored.

### 5.2. NADP(H):quinone oxidoreductase 1

Six consensus mutations (A27V, A28E, A64E, H80R, S140N and E247Q) were shown to stabilize in vitro WT NQO1, with the largest stabilization exerted by H80R [21]. This mutation alone was able to fully counteract the destabilizing effect of P187S against thermal denaturation [21]. H80R caused a local structural switch that stabilized the loop 58–67, increasing the affinity of WT NQO1 for FAD and partially counterbalancing the effect of P187S on FAD binding by dynamic remodeling of this loop [21]. Interestingly, as previously noted for Q23R and D52E compensatory mutations in AGT, H80R mediates its stabilizing effect through the reinforcement of existing electrostatic networks [21,47]. Additional structural and thermodynamic analyses supported that the enhanced affinity for FAD due to H80R was caused by subtle structural and dynamic rearrangements in the FAD binding site, consistent with a population shift in the conformational ensemble of P187S towards binding competent states ([47], according to Figure 2, a shift towards NS). However, the stabilizing effect of H80R was not efficiently propagated to the distal CTD, thus not protecting P187S against UDP degradation, but enhancing its proper folding and activity in cultured cells [21]. These results with H80R are remarkable because they highlight the potential uncoupling between conformational (in vitro) and intracellular stabilities. In contrast, the E247Q mutation located in the CTD and thus, far from the FAD binding and the His/Arg80 sites, increased the stability of the CTD but also improved FAD binding, supporting the efficient propagation of its stabilizing effect through long distances. Consistently, a double mutant H80R/E247Q showed synergistic effects on the NTD and CTD, largely abolishing the pathogenic effects of P187S through these two mechanisms [21]. Therefore, for NQO1, the local stabilizing effect of two compensatory mutations separated by 40 Å can correct different pathogenic effects in part due to the efficient propagation to distant functional sites (again, in the context of Figure 2). This clearly indicates that to understand how compensatory mutations operate, we must also consider long-range effects, not only short-range ones [20].

As indicated above for AGT, most mammalian NQO1 enzymes contain the six consensus amino acids shown to stabilize the human enzyme, including rat NQO1. As found for AGTs, the effect of consensus mutations in human NQO1 recapitulate those naturally found for consensus amino acids rat NQO1 [21]. Calculations performed with FoldX also supported that consensus amino acids could counterbalance the effects of P187S (Figure 6B), in agreement with experimental results [21]. Conversely, similar analyses by FoldX performed on the rat enzyme suggested that the destabilizing effect of P187S on this orthologue could be counterbalanced by the presence of consensus amino acids (Figure 6B), thus, according to our interpretation, making the rodent enzyme resistant against the pathogenic P187S variation.

### 5.3. On the Different Sensitivity of Human and Non-Human Mammalian Enzymes against Disease-Associated Missense Mutations

The analyses described so far with NQO1 and AGT suggest that recent divergence (over the last 50 Myr) in key consensus amino acids may have rendered these two proteins more vulnerable against disease-associated mutations. Similar consensus analyses, not yet experimentally verified, point to a similar scenario for human UDP-glucose 4-epimerase (GALE) (Figure 5), a metabolic enzyme associated with LOF inherited galactosemia due to structural destabilization and altered protein dynamics [78,79,80]. It must be emphasized that divergence from the consensus state at these sites in human NQO1 and AGT had no large effects on many traits of these proteins, including intracellular activity, folding, stability, trafficking or protein:protein interactions [21,74]. Therefore, recent loss of these consensus amino acids might have occurred through at least two plausible mechanisms [21]: (i) these sites might have been under low selective pressure, thus constituting neutral networks (i.e., sets of neutral mutations occurring in a similar evolutionary time scale; see Figure 6). The concomitant decrease in conformational stability (and to a lesser extent, of activity) due to these mutations might still provide a sufficiently functional, long-lived and well-behaved protein for adequate homeostasis; (ii) mutations diverging from the consensus might have fine-tuned certain properties in these proteins in their corresponding cellular context (note that these properties have not been identified yet).

It could be argued that the role of consensus amino acids in the different sensitivity of mammalian orthologues against disease might be a rare situation (let’s say, only for AGT and NQO1). However, it is well known that some mammalian species are particularly more resistant against disease-associated mutations than humans, which is likely associated to small divergences in the sequence and properties of the corresponding proteins [19,20,81,82]. Without a need to invoke inter-species differences in gene expression, regulation or protein homeostasis capacities, this situation has been investigated at the protein (sequence) level in this and other grounds, and referred to as compensated pathogenic deviation (CPD). A CPD just describes a situation in which a given allele is deleterious in one protein sequence while it is neutral in an orthologue sequence [10]. Noteworthy, large-scale comparative genomic analyses have estimated that the occurrence of CPD in human diseases may account for as much as 10% of disease-associated alleles overall [10]. A significant fraction of these compensatory effects leading to different interspecies sensitivity against disease seem to arise from local stability changes around the disease-associated site [20]. As we have presented here for NQO1, long-range and dynamic effects of compensatory mutations also deserve further investigation.

## 6. Post-Translational Modifications: Potential Roles in Disease and Epistatic Interactions with Pathogenic and Compensatory Mutations

Post-translational modifications provide efficient mechanisms to control protein activity, stability, and solubility in rapid response to environmental changes. Due to the advance of high-throughput proteomic analyses and efforts to characterized putative PTM sites (particularly through sequence analyses), we are realizing how much is unknown about the particular effects of PTMs on protein function and stability in the cellular environment. Although the exact number of different PTMs is not known, it is estimated to be over 200 (from the UniProt database; http://www.uniprot.org/). Since PTMs change the chemical nature of the modified amino acid, they represent a sort of expansion of the amino acid repertoire encoded by genes. For sake of illustration, we have accessed the PhosphositePlus website (https://www.phosphosite.org [83]) to provide some insightful statistics. This database contains records for over non-redundant 417,000 PTMs in 20,300 non-redundant proteins (with particularly detailed comparison between human and rodent enzymes). Remarkably, less than 5% of these sites have been characterized by site-specific methods, and thus, our knowledge for the effects on the function and stability of proteins is very scarce. About 70% of these sites correspond to Ser, Thr or Tyr phosphorylation, while about 23% correspond to either ubiquitylation or acetylation of Lys residues. The rest of PTMs are a minority (of about 30,000 sites!) formed by methylations, SUMOylations, succinylations, and O-Glc/GalNac additions. Interestingly, regarding disease-associated proteins, only those associated with common diseases have been investigated in detail: for instance, p53, p16, BRAF, or α-synuclein have about 70% of the sites investigated in a site-specific manner. However, in rare diseases such as primary hyperoxaluria type I (AGT), CBS-deficient homocystinuria (CBS), congenital erythropoietic porphyria (UROS), galactosemias (GALT, GALE), transthyretin amyloidosis (TTR), phenylkenoturia (PAH), and ACADM (acyl-CoA dehydrogenase medium chain) or ACC (acetyl CoA carboxylase) deficiency (ACADM/ACC1), the number of sites investigated by site-specific methods drops to ~5% on average.

PTMs could be fundamental to improve our understanding of LOF genetic diseases, their genotype-phenotype correlations and how evolutionary divergence at certain sites (particularly those affecting the consensus state or other epistatic interactions) may have contributed to explain different phenotypes among mammalian species carrying a given disease-associated mutation. We will illustrate these plausible relationships, again using human NQO1 and AGT.

**Figure 7:**
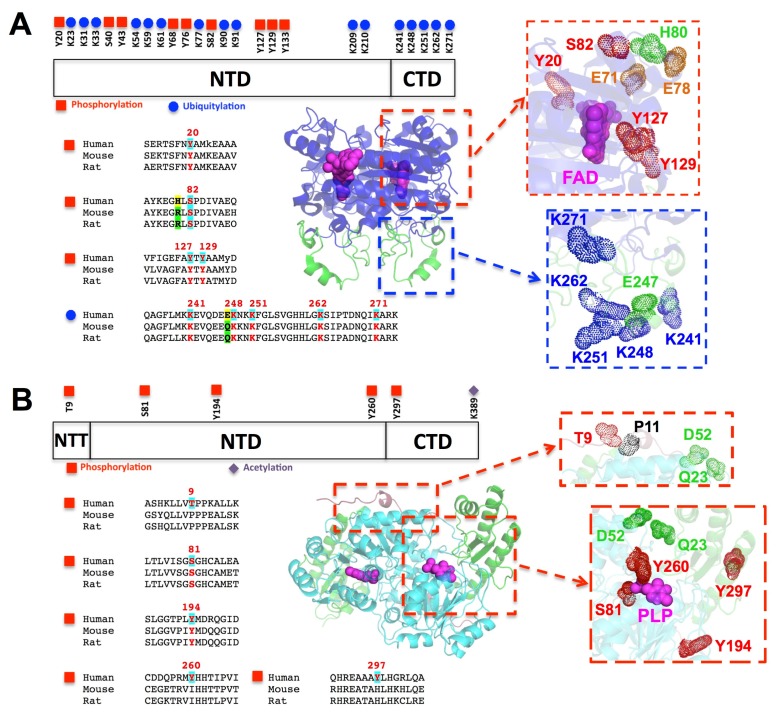
Post-translational modification (PTM) sites in human NQO1 (**A**) and AGT (**B**) as compiled in the PhosphositePlus database. The position of the PTM sites along the protein sequences and sequence alignment with mouse and rat enzymes to show their conservation are shown in the left. In the middle and right side, the location of the PTM sites, relevant disease-associated and consensus amino acids are shown. Details can be found in the text. NTD, N-terminal domain.

NQO1 contains 25 sites reported as active for PTMs (Figure 7A), 16 corresponding to ubiquitylation, and nine to phosphorylation. None of them have been studied in terms of their effects on protein function or stability. All phosphorylation sites are found in the N-terminal half of the protein. Considering our increasing knowledge on mutational effects on NQO1, and particularly the critical role of electrostatic interactions in modulating its conformation, dynamics and functionality, these phosphorylation events likely affect NQO1 activity. It must be said that electrostatic interactions are particularly suited to cause long-range perturbations because electrostatic energies decrease as a function of the distance between charges. We will discuss in more detail four phosphorylation sites. Phosphorylation at Tyr20 and Ser82 have been systematically and consistently reported (summing up to 30 different high-throughput studies), and are located in the vicinity of the FAD binding site. In particular, phosphorylation of Ser82 would introduce a negative charge close to the loop 58–67 and the EER cluster (His/Arg80, Glu71 and Glu78; Figure 7A), which are important for FAD binding, and the manifestation of LOF due to P187S and its rescue by the compensatory mutation H80R [21,47]. Therefore, phosphorylation of Ser82 might have important effects on the function and stability of WT and P187S, and also in the evolutionary divergent modulation of their phenotypes by the H80R and E247Q mutations. Phosphorylation of Tyr127 and 129, although more rarely reported, are likely critical for NQO1 function. These tyrosines establish direct intermolecular contacts with FAD and the substrate/inhibitors (Figure 7A). Regarding the ubiquitylation sites, we will discuss in more detail those found in the CTD (over 30% of the sites reported in less than 10% of the protein sequence). The five lysines found to be ubiquitylated in the CTD form part of a strong and favorably interacting electrostatic network (an estimation using the calculation previously performed in [21] gives a value of about −4 kcal·mol^−1^ for the electrostatic interaction energy), and they are likely involved in the UDP degradation of NQO1. In WT NQO1, removal of FAD leads to destabilization and increased dynamics of the CTD [54,55], which increases its ubiquitylation by CHIP (C terminus of Hsc70-interacting protein) and UDP degradation of the enzyme [56]. In the case of P187S, even the holo-state contains a partially unstructured and dynamic CTD associated with ubiquitylation and UDP degradation, which are prevented upon folding of the CTD due to binding of dicoumarol or binding of specific antibodies to this domain [54,55,56]. It is interesting to note that these five lysine residues at the CTD are conserved in mouse and rat NQO1, but no studies have reported their ubiquitylation in the rodent enzyme so far.

PTMs in AGT are less frequently detected than in NQO1, with most of them involving phosphorylation events (Figure 7B). These sites can be divided in two groups corresponding to presumable effects on stability, dimerization and function. In the first group, Thr9 is located in the extended NTT that grabs the adjacent monomer, a region important for stability and dimerization of AGT [72] (Figure 7B). This phosphorylation site seems to appear only in the sequences of *Hominoidea*, suggesting that this regulatory mechanism might be very recent in an evolutionary time scale and likely associated with changes in the subcellular location of AGT between some primates and other mammals ([62], Figure 7B). Remarkably, changes in conformational propensity of the NTT due to phosphorylating Thr9 would also affect the transition towards non-native helical structures induced by the P11L polymorphism in this region [84]. It is also worth noting, that the negative charge introduced upon phosphorylation of Thr9 may cross-talk with local electrostatic changes introduced by the consensus mutations Q23R and D52E (Figure 7B). In a second group, phosphorylation of Ser81 and Tyr260, part of the PLP binding site, will certainly have important effects on catalysis, structure and stability (Figure 7B). The effects of phosphorylating Tyr194 and Tyr297 would be milder, but still they are in the proximity of the PLP binding site (Figure 7B). The introduction of negative charges upon phosphorylation of Tyr260 and Tyr297 might also interact with the consensus mutation Q23R, in relative proximity to them.

## 7. Concluding Remarks

Conformational diseases are an enormous burden for our society. The lack of efficient therapies for these diseases make development of novel therapeutic strategies an important task. The understanding of the mechanisms by which disease-associated mutations lead to protein LOF are fundamental to achieve this task. However, the link between mutational effects on protein structure, stability and dynamics with the behavior of the protein inside the cell, where it interacts with the protein homeostasis network, is a remarkable scientific challenge. In these regards, studying compensatory mutations may help decipher disease mechanisms and identify structural hot spots for therapeutic intervention, revealing how different structural and functional sites in proteins are functionally and energetically coupled in the native ensemble, perturbed by disease-associated mutations and potentially corrected by pharmacological agents. In addition, the use of compensatory mutations as adaptive tools to develop novel protein and nucleic acid based therapies are beginning to yield promising results (Figure 5 and [75]) and deserve further research. Linked to this understanding of disease-mechanisms, inter-species changes in compensatory mutations may also provide information on the mechanisms of protein evolution, particularly into epistatic mechanisms at the protein level [10,19,85]. Of particular relevance, detailed analyses of the effects of disease-associated and compensatory mutations would require considering long-range structural and energetic effects, as well as their impact in protein dynamics. In addition, our understanding of all these issues will certainly improve when the effects of PTMs will be incorporated into the picture.

## Appendix A

The sequence encoding the TAT peptide (RKKRRQRRRG) was inserted at the N-terminus of the AGT-RHEAM complementary DNA (cDNA) cloned in pET43(1a) vector by subsequent steps of insertion polymerase chain reaction (PCR) using the primers reported in [76]. The TAT-AGT-RHEAM and TAT-AGT constructs were then transformed in *Escherichia coli* BL21 cells and the two proteins were expressed and purified as previously reported [76].

CHO-GO cells are considered an excellent cellular model of PH1 because they recapitulate the main features of liver glyoxylate metabolism. Cells were cultured at 37 °C under O_2_/CO_2_ (19:1) atmosphere in Ham’s F12 Glutamax medium (Invitrogen, Carlsbad, CA, USA) supplemented with fetal bovine serum (10%, *v*/*v*), penicillin (100 units/mL), streptomycin (100 μg/mL), and Zeocin (400 μg/mL). To define the time-course of TAT proteins internalization, 2 × 10^5^ CHO-GO cells were seeded in 3 cm diameter culture dishes, and treated with 1.5 µM TAT-AGT-RHEAM. At various incubation times (0, 4, 8, 24, 48 h), cells were harvested and kept at −80 °C until lysis. As negative control, CHO-GO cells were treated with AGT-RHEAM 1.5 µM and incubated for 48 h. CHO-GO cells stably expressing AGT (CHO-AGT) were used as a positive control. Cells were lysed as previously described [86] and subjected to sodium dodecyl sulfate-polyacrylamide gel electrophoresis (SDS-PAGE), transfer on nitrocellulose membrane, incubation with anti-AGT (Rb) 1:6000, and detection by chemiluminescence.

For dose-response curves, 6 × 10^5^ cells were seeded in 6 cm diameter dishes and treated with various concentration of TAT-AGT-RHEAM (0.1, 0.5, 1, 1.5, 3, 7.5, 15 µM) or with 7.5 µM TAT-AGT for comparison. Twenty-four hours after treatment cells were harvested, lysed as previously indicated and analyzed for protein expression level and transaminase activity. As positive and negative control, CHO-AGT cells and CHO-GO cells treated with 15 µM AGT-RHEAM were analyzed. The transaminase activity of CHO-GO cells was assayed by incubating 100 μg of soluble lysate with 0.5 M l-alanine and 10 mM glyoxylate for 30 or 60 min in the presence of 200 μM PLP. The reactions were stopped by adding trichloroacetic acid (TCA) 10% (*v*/*v*). Pyruvate formation was measured by a coupled spectrophotometric assay described in [87].

The indirect glycolate toxicity assay was performed by incubating CHO-GO cells at 8000 cells/well in a 96-well plate with TAT-AGT-RHEAM, TAT-AGT or AGT-RHEAM (7.5 μM). After 24 h, glyoxylate production was induced by adding HEPES-buffered glycolate, pH 7.0, at a final concentration of 0.5 mM. CHO-AGT and untreated CHO-GO cells were used as positive and negative control, respectively. Cell viability was evaluated after further 24 h incubation using the crystal violet staining. Briefly, cells were rinsed with PBS (phosphate buffered saline) and incubated at room temperature for 5 min with 4% formaldehyde +0.5% crystal violet solution to perform fixing and staining. Cells were then extensively washed with distilled water to remove the excess of dye and lysed with 1% SDS in PBS to allow crystal violet solubilization and quantification. The absorbance at 595 nm, which is proportional to the number of viable cells, was measured with a NanoQuant Infinite M200PRO (Tecan, Männedorf, Switzerland) plate reader. Eight replicates were performed for each assay condition.

## Appendix B

Unfolding Gibbs free energy changes (∆∆G) between a mutant and a reference were estimated by using the FoldX algorithm (FoldX 4 version) in order to obtain a structure-based evaluation of the effect of mutations on the protein stability [77]. In this algorithm, ∆∆G is calculated between the tridimensional structure (pdb file) and a hypothetical unfolded state by using a properly weighted linear combination of empirical terms (solvent interaction, hydrogen bonds, electroctatic contribution, etc). Stability calculations with FoldX reproduce experimental values with an uncertainty of about 1 kcal·mol^−1^ [39,77,88].

The crystal structure of each protein was used as the main input for these calculations (human AGT: PDB 1H0C; mouse AGT: PDB 3KGX; human NQO1: PDB 2F1O; and rat NQO1: PDB 1QRD). Mutations were inserted progressively as indicated by the *x*-axis of Figure 6, thus evaluating the changes for each batch. All structures were subjected to the same analysis by applying the following three steps: (1) Reduction of energy: Although high resolution crystal structures represent a low energy state of the protein, in this first step, the global energy of the structure is optimised by using the FoldX “RepairPDB” command; (2) Introduction of mutation/s batch: selected mutations were introduced and the ∆∆G values (in kcal·mol^−1^) were obtained at 298 K, pH 7, ionic strength 100 mM and Van der Waals design set to default by using the FoldX “BuildModel” command. Finally, when needed, the resulted structure was saved as a new pdb file to be used as an input for the next step.
